# Analysing the global and local spatial associations of medical resources across Wuhan city using POI data

**DOI:** 10.1186/s12913-023-09051-0

**Published:** 2023-01-28

**Authors:** Qiao Chen, Jianquan Cheng, Jianguang Tu

**Affiliations:** 1 School of Tourism and Hospitality Management, Hubei University of Economics, Wuhan, 430205 China; 2 Department of Natural Sciences, Manchester Metropolitan University, Manchester, M1 5GD UK; 3 Centre for Health Geographic Information, Key Laboratory of Environmental Change and Resource Use in Beibu Gulf (Ministry of Education), Nanning Normal University, 175 MingxiuDonglu Road, 530051 Nanning, PR China; 4 School of Remote Sensing and Information Engineering, Wuhan University, Wuhan, 430070 PR China; 5 China Aero Geophysical Survey and Remote Sensing Center for Natural Resource, Beijing, 100083 China

**Keywords:** Medical resource, Spatial association, Localized colocation quotient, Wuhan

## Abstract

**Background:**

There is a sharp contradiction between the supply and demand of medical resources in the provincial capitals of China. Understanding the spatial patterns of medical resources and identifying their spatial association and heterogeneity is a prerequisite to ensuring that limited resources are allocated fairly and optimally, which, along with improvements to urban residents’ quality of life, is a key aim of healthy city planning. However, the existing studies on medical resources pattern mainly focus on their spatial distribution and evolution characteristics, and lack the analyses of the spatial co-location between medical resources from the global and local perspectives. It is worth noting that the research on the spatial relationship between medical resources is an important way to realize the spatial equity and operation efficiency of urban medical resources.

**Methods:**

Localized colocation quotient (LCLQ) analysis has been used successfully to measure directional spatial associations and heterogeneity between categorical point data. Using point of interest (POI) data and the LCLQ method, this paper presents the first analysis of spatial patterns and directional spatial associations between six medical resources across Wuhan city.

**Results:**

(1) Pharmacies, clinics and community hospitals show “multicentre + multicircle”, “centre + axis + dot” and “banded” distribution characteristics, respectively, but specialized hospitals and general hospitals present “single core” and “double core” modes. (2) Overall, medical resources show agglomeration characteristics. The degrees of spatial agglomeration of the five medical resources, are ranked from high to low as follows: pharmacy, clinic, community hospital, special hospital, general hospital and 3A hospital. (3) Although pharmacies, clinics, and community hospitals of basic medical resources are interdependent, specialized hospitals, general hospitals and 3A hospitals of professional medical resources are also interdependent; furthermore, basic medical resources and professional medical resources are mutually exclusive.

**Conclusions:**

Government and urban planners should pay great attention to the spatial distribution characteristics and association intensity of medical resources when formulating relevant policies. The findings of this study contribute to health equity and health policy discussions around basic medical services and professional medical services.

## Introduction

Since the introduction of economic reform and the open-door policy in 1978, China has established a programme of rapid urbanisation, experiencing the fastest rate of economic growth in the world; however, this has been done at the cost of significant environmental degradation. The growth of China’s ageing population, as well as the related increased health burdens [1], has created new challenges for urban sustainability. In 2019, one-fifth of the population (1.94 m people) in the Wuhan municipality was aged 60 and above. Of these individuals, 2.9% (261,200 people) were senior citizens aged ≥ 80, representing an increase of 10,300 since 2018 [2]. To meet the needs of the growing ageing population, the municipal government is investing heavily in medical resources; however, to address the spatial inequality in residents’ accessibility to these medical resources, additional quantitative evidence on their spatial patterns is needed [3, 4]. As such, policy-makers are concerned with the following questions: what are the key characteristics of the spatial distribution of medical resources? What spatial associations exist between different categories of medical resources? The availability of point of interest data, which can be freely captured from the internet using data crawling techniques [5–7], means that the methods presented in this study can be easily replicated in other cities.

In the current literature, the majority of studies on medical resources have focused on three aspects. The first aspect is analysis of the spatial distribution characteristics between medical resources and community residents, such as the spatial distribution characteristics of various health resources in cities [8], the inequality of resource distribution [9], the spatial configuration of medical resources at different levels in different regions [10], and the supply mode and accessibility to medical resources [11]. Such studies frequently use regression models, such as ordinary least squares regression (OLS) [12], geographically weighted regression (GWR) [13, 14], and logistic regression (LR) [15], to model and explain spatial patterns. The majority of the studies use aggregate data from national censuses, cross a range of scales from subdistrict to community [16, 17]. Geographically weighted analytical methods, including regression and principal component analysis, have been proven effective and efficient in dealing with spatial heterogeneity, specifically spatial nonstationarity. However, GIS (Geographic Information System) applications for these methods, commonly used for urban governance or management, do not analyse individual spatial interactions. For example, changing the spatial extent of the study area in a GIS analysis can create multiscale effects, which have been highlighted in many analytical methods, such as GWR [18], logistic regression [19, 20], spatial autocorrelation analysis [21, 22], cluster analysis [23], and spatial differentiation [24].

The second aspect is analysis of the spatial association between medical resources and community residents. Increasing evidence has confirmed that urban residents’ medical-seeking behaviour is highly influenced by their spatial accessibility to medical facilities [25]. Specifically, people living in central cities have easier access to subway stations and consequently better accessibility to hospitals at different levels. In contrast, residents living in suburban areas demonstrate a high dependence on community hospitals. Only when community hospitals are farther away than general hospitals do these residents show a strong preference for general hospitals. When both categories of hospitals are not accessible, they tend to self-treat themselves by purchasing medicines from local pharmacies [26]. It is clear that residents’ medical treatment behaviour is highly dependent on the trade-off between medical service quality, transport accessibility, and the spatial distribution of medical facilities and social systems.

In recent years, the growth of big data technology has increased the availability of point data. Relevant studies mainly focus on the following two points: (1) Spatial associations and heterogeneity of categorical Point of interest (POI) big data. Point of interest data have been increasingly used for urban vibrancy [27], space of urban hotel [28], food culture [29], because of their low cost and high temporal resolution. Jiang et al. (2021) used restaurant POI data to explore the regional structure of food culture based on cuisine preference [30]. Zhang et al. (2021) examined the food culture of mid-eastern China using millions of items of restaurant point of interest data and explored different spatial patterns between local and nonlocal restaurants [29]. Zhou et al. (2023) developed an electronic word of mouth (E-WoM) index system of hotels in Nanjing by using Dianping.com data and analysed the hotel central place hierarchy based on the consumption price and E-WoM score [28]. Many POI data are available in the form of categorical rather than interval and ratio variables and require specific analytical methods suitable for measuring the spatial associations and heterogeneity of categorical point data.

The third aspect is the evolution of homotopic pattern analysis methods. In contrast to other methods for measuring spatial association (such as Moran’s I for interval and ratio data), the colocation quotient between multiple categories is particularly suitable for urban analytics. Leslie & Kronenfeld (2011) developed a global colocation quotient (GCLQ), and subsequently, Cromley et al. (2014) developed a local colocation quotient (LCLQ) by incorporating a geographical weighted method into the global colocation quotient [31]. Further advances include Wang et al.’s (2017) application of a Monte Carlo simulation to create a test of the statistical significance of the colocation quotient results. Colocation quotients are now widely used in urban studies [32]. To date, studies have focused on determining the nature of spatial associations between categorial variables, such as the colocation between trees and business establishments [33]; the spatial co-occurrences of food sources (convenience stores, fast food outlets) and pharmacies [34]; the spatial correlation between transportation infrastructure and the location of competing retail firms [35]; the spatial relationship between types of crime and facilities such as bars, schools, and shops [36]; the spatial association between types of crimes and land use [32]; the spatial correlation patterns between intersections and crashes (pedestrian- or cyclist-involved) [37]; associations between fires and land-use types [38]; crashes related to various traffic accidents (minor injury, major injury, and fatal) [39]; and the spatial associations between cycling activities and urban facilities [40]. In recent years, with the development of information technology, the related methods of colocation patterns have been further improved and expanded. Ran et al., (2022) measure the spatial proximity relationship between different types of medical resources and summarize the spatial proximity effect of medical resources [41]. Juhász et al. (2021) found both that colocated technologies are more likely to become related over time and that colocation and the complexity of technologies are conducive to intensification [42]. Zhang et al. (2022) proposed a colocation pattern detection and spatial association rule discovery approach that treats origin–destination (OD) flows as Boolean spatial features while considering the spatial proximity of the origins and destinations of OD flows and their direction similarity [43].

However, there are still some shortcomings in the published literature. To date, few studies have used the colocation quotient to measure the spatial heterogeneity or directional spatial association between types of medical resources. Most of the existing studies have used GWR, kernel density and other analytical methods to explore the spatial distribution pattern and evolution of medical resources, while few have examined the spatial co-location and dependence intensity among medical resources. By using the global and local location quotient method, this study aims to measure not only the “adjacent” and “exclusive” associations and intensity between different types of medical resources globally but also the direction and intensity of spatial dependence between the same type of medical resources locally. It is worth noting that the study on the spatial relationship and intensity between medical resources is a prerequisite for achieving the spatial equity and operational efficiency of urban medical resources. This paper explores all these issues by applying both global and local colocation quotients to medical resource POI data from Wuhan city.

This paper is organized as follows. Sect. "Materials and methods" describes the study area and data sources and then explains the global and local (geographically weighted) colocation quotient methods used. Sect. "Results" presents and interprets the analytical results. Sect. "Conclusions" offers conclusions drawn from the results and suggestions for future research.

## Materials and methods

### Study area

**Fig. 1:**
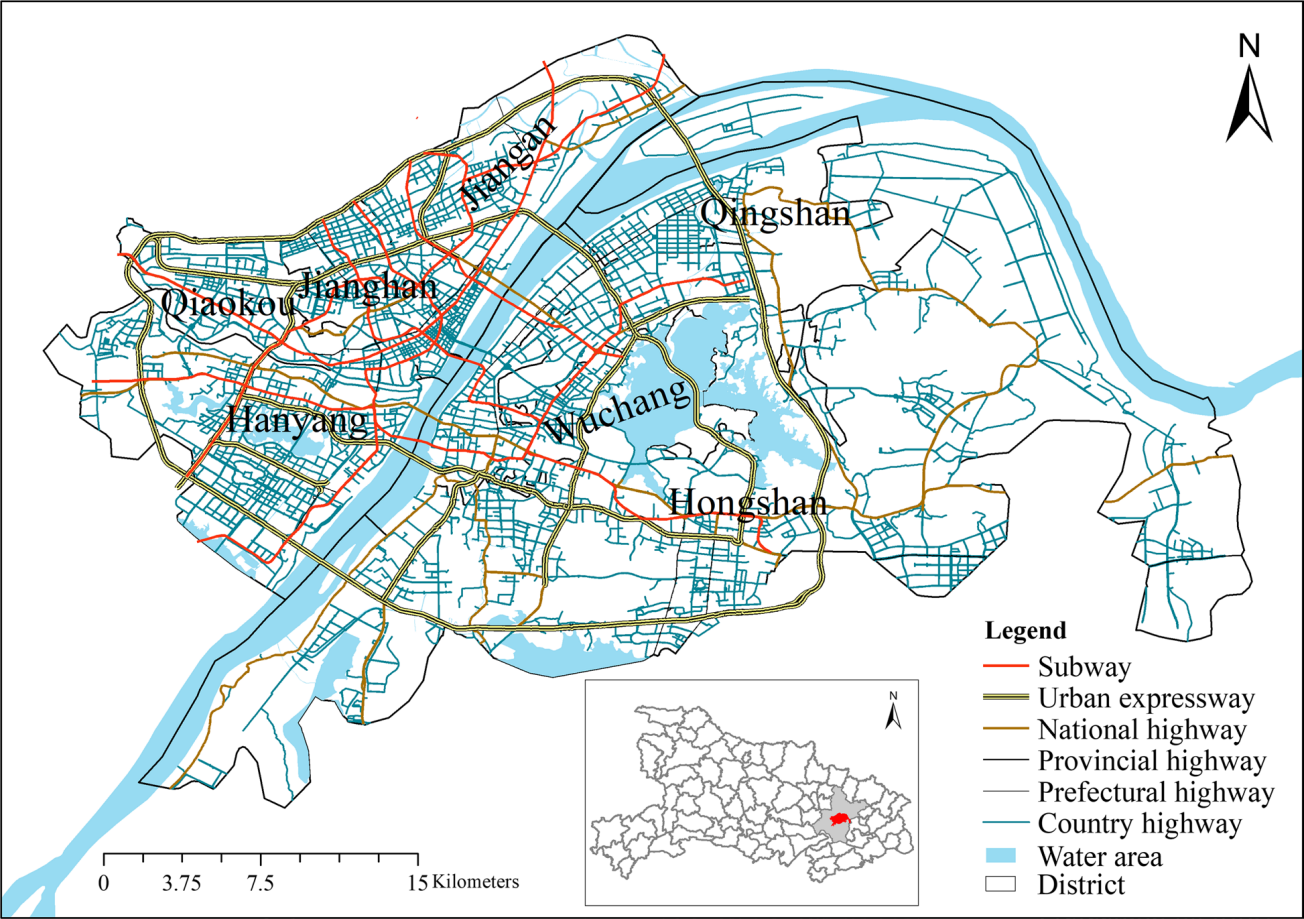
The location of the study area in central Wuhan, Hubei Province

Wuhan, the capital of Hubei Province, is located in central China between 113°41’-115°05’ E and 29°58’-31°22’ N [44]. The highest concentration of urban socioeconomic activities and residential buildings is located in the seven urban districts in central Wuhan (Fig. 1). In 2020, this area (863 km2) had a population of 11.2 million people and a GDP of 1.62 trillion RMB.

To follow the national “Healthy China 2030” strategy [45], the Wuhan municipality developed a range of policies, such as the “Healthy Wuhan 2035 Plan” in 2018 and “Wuhan People’s Government’s Implementation Opinions on Accelerating the Development of the Health Service Industry”, with the aim of improving the health and quality of life of its population [46].

### Data sources

In 2015, the General Office of the State Council issued “Guidance on Promoting the Construction of a Hierarchical Medical System”, which advocated “primary treatment at the grassroots level, two-way referrals, separation of emergency and nonurgent treatments, up and down linkage” [47]. The establishment of a hierarchical medical system aims to reverse the current unreasonable pattern of medical resource allocation and promote cooperation between various medical organisation. These social cooperation results in spatial association between these medical resources. In addition, the report of “Guiding Principles for the Planning of Medical Institutions (2021–2025)” formulated by the National Health Commission also clearly points out that governments should take scientific creativity and collaborative innovation as the basic principles [48], to strengthen the spatial dependency between medical resources, optimize the spatial layout of medical resources, and build a high-quality, balanced and efficient medical service system. The driving factors of spatial co-location between medical resources are assumed as follows:

Firstly, organizational coordination and professional cooperation (e.g., diagnosis, treatment, resource sharing) should be the primary factor of spatial association between these hospitals and clinics. The Commission of Health and Family Planning requires high-quality medical resources (3A hospitals, general hospitals, etc.) to be located within the jurisdiction. Such spatial proximity as a basic principle of site selection [49], was used to establish a medical service cooperation system with basic health and medical institutions (community hospitals, clinics, etc.) by means of pairing [50], forming medical consortiums, and remote consultation [51]. Therefore, in order to facilitate the collaboration between these medical resources, the government will strengthen the spatial co-location between medical resources when planning medical systems.

Secondly, population size might be another factor of spatial co-location between medical resources. Population size determines the demand for medical resources between different regions and levels [52]. In order to achieve medical service equity, the government should match the population demand for services and provision for services from these medical resources, for example, small-scale clinics should be located with residential neighbourhoods [53]. Thereby, the spatial agglomeration and spatial co-location between this hierarchical medical system is adjusted by population distribution.

Thirdly, transport network might be an influential factor on the spatial relationships between these medical resources. Generally speaking, medical resources are distributed in areas with high transport accessibility for visiting hospitals [54]. Large-scale medical resources (e.g. general hospitals) should be located in areas with highest level of transport accessibility, e.g. near sub-way station [41]. Thereby, spatial co-location between the medical resources could be shaped by the transport network.

**Fig. 2:**
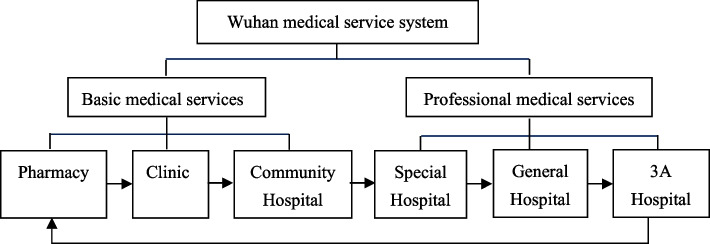
The hierarchy of the Wuhan medical service system

The complicated spatial interactions between medical resources, population distribution and transport network might be the logic behind the heterogeneous spatial associations between the hierarchical medical systems and resources [55]. Based on the concept and attributes of medical services, medical services in urban China are generally divided into two systems [56]: basic medical services, including pharmacies, clinics, and community hospitals, and professional medical services, including specialized, general and 3A hospitals^1^ (Fig. 2).

In this study, medical resources refer to the medical services shown in Fig. 2, including the personnel, buildings, beds, facilities and equipment, budget, knowledge, skills and information required to operate them [57]. Hospitals are allocated to the basic or professional services group based on service demand, scale and quality [58, 59]. Community hospitals typically provide basic disease prevention, basic treatments, and nursing, whereas specialist, general and 3A hospitals provide medical services for complex or severe diseases.The location and classification of all medical resources within the study area were captured from Amap API (https://developer.amap.com/product/map) in December 2019 using a web-crawling technique. Preliminary data processing included coordinate correction, a coordinate point reverse check, and address information correction and completion. A total of 5,401 POI data points were collected (Table 1). Each POI included the medical resource name (incloud headquarters and branches), identity number, latitude and longitude coordinates, and specific address. District boundary and road network data were also collected from the National Basic Geographic Information System database.

**Table 1:** Classification of medical resources in the study area

ID	Classification	Category	Count
1	Basic	Pharmacy	2383
2		Clinic	1548
3		Community Hospital	391
4	Professional	Specialist Hospital	737
5		General Hospital	297
6		3A Hospital	45
	Total:		5401

### Methods

#### Nearest Neighbor Indicator

Nearest Neighbor Indicator (NNI), first proposed by Clark and Evans [60], was applied to plant ecology, and then introduced by King to explore the spatial distribution pattern of urban settlements. This method mainly calculates the average distance between each point and its nearest neighbor point (Eq. 1). The nearest neighbor index reflects the clustering and dispersion degree of the whole point distribution.

1 $$\mathrm{NNI}=\frac{\sum_{i=1}^{n}\frac{\mathrm{min}({d}_{ij})}{n}}{0.5\sqrt{\frac{A}{n}} }$$

where $NNI$ is the nearest neighbor analysis coefficient, A is the area of the study area, n is the number of sample points, ${\mathrm{d}}_{\mathrm{ij}}$ is the distance from point i to point j. When $NNI$ > 1, the distribution of points is uniform and dispersed; When $NNI$ = 1, points are randomly distributed; When $NNI$ < 1, the spatial distribution of points is clustered. Z-test is mainly used to test the statistical significance of such point pattern. If Z < -2.58, the points demonstrate a clustered pattern at 99% confidence level or 1% significance level.

#### Global colocation quotient

To analyse the colocation patterns of various point sets, Leslie & Kronenfeld (2011) proposed the global CLQ, which measures the overall extent to which category A points (e.g., one type of medical resource) are dependent on category B points (e.g., another type of medical resource) [32]. In contrast to other methods such as Moran’s I, global CLQ uses nearest neighbours rather than Euclidean distance to quantify the spatial association between different sample populations. Where a point has multiple nearest neighbours within a bandwidth, each nearest neighbour is assigned an equivalent weight, as shown in Eq. 2:

2 $${N}_{A\to B}=\sum_{i=1}^{{N}_{A}}\sum_{j=1}^{n{n}_{i}}\frac{{f}_{ij}}{n{n}_{i}}$$

where $i$ is category A, ${\mathrm{nn}}_{\mathrm{i}}$ denotes the *i*th nearest neighbour, and $j$ is the number of nearest neighbours to point $i$. ${f}_{ij}$ is a binary variable indicating whether point A’s nearest neighbour $j$ is type B (1 means yes, 0 means no). ${N}_{A\to B}$ denotes the number of type A points that have type B points as their nearest neighbours. The global CLQ is calculated using Eq. 3:

3 $${GCLQ}_{A\to B}=\frac{{N}_{A\to B}/{N}_{A}}{{N}_{B}/(N-1)}$$

where ${N}_{A}$ and ${N}_{B}$ represent the number of type A and B medical resources, respectively, and ${N}_{A\to B}$ is the number of type A points (Eq. 2), whose nearest resource is type B. $\mathrm{N}$ is the total number of medical resources (5401) in the study area (Table 1). When calculating expectations, the denominator is (N-1) instead of $\mathrm{N}$ because a point cannot be its own neighbour [36].

${\mathrm{GCLQ}}_{A\to B}$ measures the extent to which type A points are attracted to type B points [33]. When ${\mathrm{GCLQ}}_{A\to B}=1$, type A and B points are both randomly distributed within the study area. That is, the proportion of type B nearest neighbours to type A points is equal to the overall proportion of type B points in the sample. When ${\mathrm{GCLQ}}_{A\to B}>1$, category A is dependent on B, and the number of nearest neighbours of type B is higher than expected. The greater the value is, the more dependent it is. When ${\mathrm{GCLQ}}_{A\to B}<1$, A and B are mutually exclusive, that is, the number of type B nearest neighbours is lower than expected. The smaller the value is, the greater the spatial independence between the two types. The smallest possible value of ${\mathrm{GCLQ}}_{A\to B}$ is zero, which indicates that there are no type B nearest neighbours to type A points. The spatial association expressed by ${\mathrm{GCLQ}}_{A\to B}$ is unidirectional because A and B may have an asymmetric nearest neighbour dependency. If A’s nearest neighbour is B and B’s nearest neighbour is not A, then ${\mathrm{GCLQ}}_{A\to B}$>${\mathrm{GCLQ}}_{B\to A}$, which indicates that the spatial attraction of B to A is greater than that of A to B.

#### Local colocation quotient

The local colocation quotient (LCLQ) developed by [31] was used to analyse the spatial heterogeneity of medical resources and map clusters of points with high spatial associations. Compared with the global colocation quotient, the LCLQ produces maps that are easier to interpret [36]. LCLQ is calculated as follows in Eq. 4:

4 $${LCLQ}_{{A}_{i}\to B}=\frac{{N}_{{A}_{i}\to B}}{{N}_{B}(N-1)}$$

where $\mathrm{N}$ and ${\mathrm{N}}_{B}$ are the same as those explained in Eq. 3,. ${N}_{{A}_{i}\to B}$ represents the geographically weighted average of type B points within the bandwidth of type A points, as shown in Eq. 5:

5 $${N}_{{A}_{i}\to B}=\sum_{j=1(j\ne i)}^{N}\left({w}_{ij}{f}_{ij}/\sum_{j=1(j\ne i)}^{N}{w}_{ij}\right)$$

6 $${w}_{ij}=\mathrm{exp}\left(-0.5*\frac{{d}_{ij}^{2}}{{d}_{ib}^{2}}\right)$$

${A}_{i}$ represents the i-th type A point, ${f}_{ij}$ indicates whether the nearest neighbour to point ${A}_{i}$ is point B, then ${f}_{ij}$=1, or if not ${f}_{ij}$= 0, ${w}_{ij}$ is the weight of point j, indicating the importance of individual $j$ to individual ${A}_{i}$. ${d}_{ij}$ represents the distance between point ${A}_{i}$ and point $j$, and ${d}_{ib}$ represents the bandwidth of the search area for neighbours of ${A}_{i}$. The Gaussian kernel density function in Eq. 6 was used to assign the geographical weight value to each neighbour of ${A}_{i}$. The farther each neighbour is from ${A}_{i}$, the smaller its weight will be. An adaptive bandwidth is typically used for study areas with heterogeneous density, e.g., one containing both urban and rural areas. A bandwidth determined by distance rank guarantees that each type A point has exactly the same number of neighbours, making the results more robust and reliable [36]. The local location quotient (LCLQ) is expected to be 1. Therefore, if ${\mathrm{LCLQ}}_{{A}_{i}\to B}$ is greater than 1, it indicates that ${A}_{i}$ is nearest to a type B point. The greater the value is, the stronger the association between types is. Conversely, if ${\mathrm{LCLQ}}_{{A}_{i}\to B}$ is less than 1, ${\mathrm{A}}_{i}$ does not have a type B point as its nearest neighbour. A Monte Carlo simulation was repeated 1000 times to test the statistical significance of the local location quotient results [36].

## Results

### Spatial pattern of medical resources

**Fig. 3:**
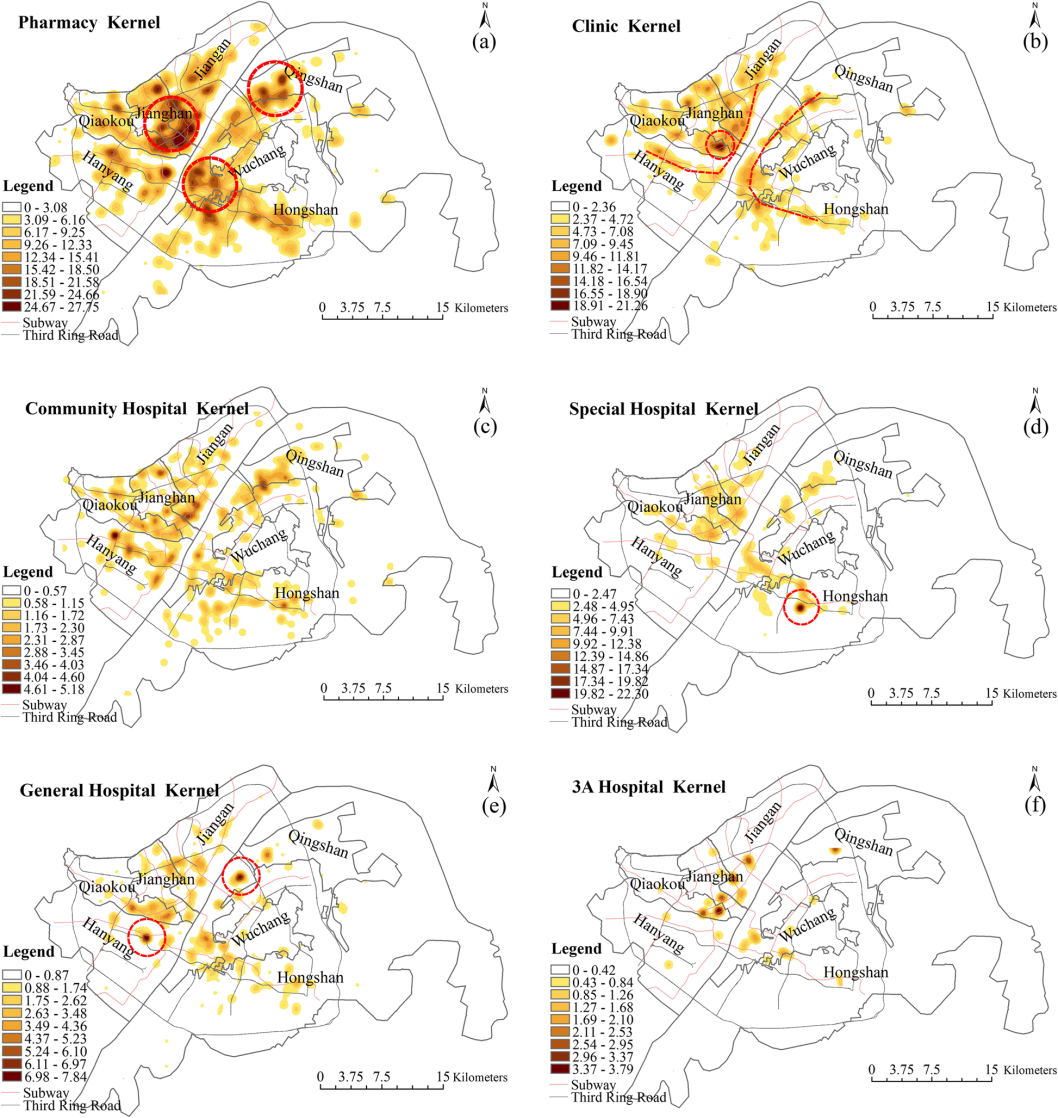
Kernel density estimation of **a** Pharmacies; **b** Clinics; **c** Community Hospitals; **d** Special Hospitals; **e** General Hospitals; and **f** 3A Hospitals

The kernel density analysis indicated the high concentration and clustering of all types of medical resources across the city centre, bisected by the Yangtze River (Fig. 3).

Pharmacies are relatively widely distributed within the study area, with three high-density clusters represented by the red circles in Fig. 3a. Pharmacies are the most widely used medical resource at the community level in China, as there is no need for a doctor’s prescription to purchase the majority of medicines [61]. Because pharmacies are usually open from 7 am-11 pm and typically located within a 15-min walking distance from residential buildings [62], the distribution of pharmacies is relatively dense and mainly concentrated in residential areas. Clinics are distributed along the banks of the Yangtze River, with an area of high density in the Qiaokou district (Fig. 3b). The distribution of community hospitals followed a similar pattern, although the areas of highest density were located in Qingshan district, Hanyang district, Jiangan district and other former urban areas (Fig. 3c). Community hospitals were the most evenly distributed of all medical resources across the city centre and suburban areas. This reflects the boundaries of the smallest administrative unit to which medical resources are allocated in China’s medical system.

Specialized hospitals form the only high-density spot in Huquan Street, Hongshan district. Huquan sub-district in Hongshan district has the highest concentration of specialized hospitals for several reasons: This sub-district is home to many large-scale universities and colleges with a roughly estimated total of 100, 000 students, including Wuhan University, Central China Normal University, Hubei Water Resources and Hydropower Vocational and Technical College, and Hubei Communications Vocational and Technical College. In addition, this area also has the high concentration of socio-economic activities, due to large-scale shopping and entertainment streets including Huquan Night Market and Mouse Street. The subway Line 2 passing through this sub-district, has provided high-level transport accessibility to these campuses and streets. These high-density land uses and accessible transport infrastructure have attracted massive flows of people, who have become the driving forces of specialized hospitals such as stomatology and medical cosmetics, which are particularly demanded by young people.Most districts are characterized by a scattered distribution of special hospitals following the subway line. This also suggests that accessibility is a key factor in siting special hospitals (Fig. 3d). The high-density areas of general hospitals are concentrated in Wuchang and Hankou districts, presenting a “dual-core” pattern on either side of the Yangtze River (Fig. 3e). The 3A hospitals are mainly concentrated in the three former districts of Jiangan, Jianghan and Qiaokou (Fig. 3f). Newer districts only have branches or subunits of these hospitals, such as the Tongji Hospital Guanggu branch and Union Hospital Jinyinhu branch.

These spatial patterns suggest a degree of inequality, where high-level medical resources are excessively concentrated in central urban areas. Due to the influence of the historical development of Wuhan, the old urban area still has the highest concentration of high-level medical resources. The patterns also show the lack of high-level medical resources in the new urban areas around the city centre. This may reflect the sparse residential areas and low population density in the new urban areas, which lead to insufficient provision and allocation of high-level medical resources.

**Fig. 4:**
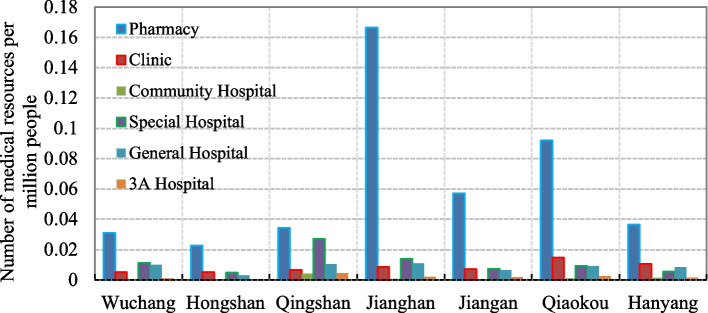
Distribution of medical resources between the seven urban districts in Wuhan

The results further show the great disparity in the spatial distribution of various medical resources between the two sides of the river. By calculating the number of medical resources per capita per unit area of each district (Fig. 4), it was found that pharmacies are mainly distributed in Jianghan district, special hospitals are mainly distributed in Qingshan district, and clinics are mainly distributed in Qiaokou district. There is a relatively small spatial disparity in the distribution of community hospitals, general hospitals and 3A hospitals. This may reflect the fact that the site selection, investment and construction of these resources are determined by the central government [63], while pharmacies, clinics and specialist hospitals are mostly privately owned. To improve the equality and equity of public medical resource distribution, the government should distribute medical resources in accordance with the current population distribution and demographic structure across the city. There are obvious differences in the form, pattern and quantity of pharmacies, clinics and special hospitals, which suggests that the coordination of different resources in Wuhan is still inadequate.

Finally, As shown in Table 2, The degree of agglomeration was pharmacy (0.422), clinic (0.476), community hospital (0.478), special hospital (0.547), general hospital(0.626) and 3A hospital(0.732). Because of historical inertia and living habits, pharmacies are mainly distributed around residential areas, the space distribution of pharmacies are the most concentrated and have strong aggregation characteristics. In addition, the nearest neighbor indicator of community hospitals, general hospitals and 3A hospitals was lower than that of specialized hospitals. It is mainly related to the public characteristics of the community hospitals, general hospitals and 3A hospitals. Their layout is often regulated by the government’s planning, and more attention is paid to the balance of spatial layout, so as to ensure every resident has the opportunity to enjoy basic medical resources.

**Table 2:** NNI of medical Resources in Wuhan

Variable	Pharmacy	Clinic	Community Hospital	Special Hospital	General Hospital	3A Hospital
NNI	0.422	0.476	0.478	0.547	0.626	0.732
Z score	-52.83	-39.36	-27.09	-14.82	-4.80	-10.14
*P* values	0.000	0.000	0.000	0.000	0.000	0.000

### Spatial associations

Leslie and Kronenfeld (2011) developed a tool to implement the global CLQ method (see https://ux1.eiu.edu/~bjkronenfeld/projects/) [33]. Following their approach, we set the bandwidth as the first-order neighbour (i.e., the nearest neighbour) before gradually increasing the number of neighbours. The GCLQ was calculated for each increase. A Monte Carlo simulation, repeated 1000 times, was used as a significance test. Finally, the number of neighbours (bandwidth) was determined by comparing the results. Using the same bandwidth to measure the global and local locations of all health resources can lead to biased results. To ensure robust, reliable results, this study used various bandwidths, each suited to the particular spatial distribution characteristics of the different medical resources.

#### Global location quotient analysis (GCLQ)

**Fig. 5:**
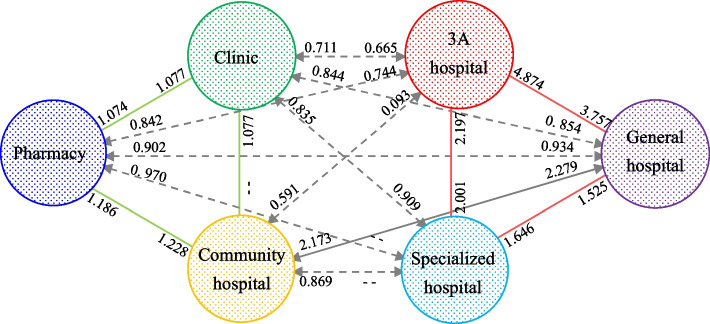
Directional spatial association between medical resource categories

The GCLQ results (Table 3) also show a degree of symmetric association—two-way colocation—between medical resource types. The GCLQ value is greater than 1 between each type of medical resource in the professional category (special hospitals, general hospitals and 3A hospitals) (Fig. 5). The strongest colocation is between 3A and general hospitals. This suggests that these resources complement each other in terms of service scale and target diseases [64, 65]. Two other pairings are evident; namely, pharmacies and clinics and pharmacies and community hospitals both exhibit some symmetric association, although the degree of two-way colocation is less than that within the professional category (Fig. 5). The significance of pharmacies in the spatial associations detected is explained by their central role in local communities, namely, providing longer opening hours than hospitals and easy access to medication [66, 67], particularly after a diagnosis from a clinic or community hospital visit. There is an asymmetric association between clinics and community hospitals, the former being dependent on the latter (GCLQ = 1.077) but the opposite not being significant. This is because there is only one community hospital per community, which means that it is likely to be located in an area with good accessibility to all residents. However, clinics, being mostly private, smaller scale services, are typically located near residential areas. As such, community hospitals do not have a significant spatial association with clinics.

**Table 3:** Global colocation quotient for all medical resources

Categories	Pharmacy	Clinic	Community Hospital	Special Hospital	General Hospital	3A Hospital
Pharmacy	——	2(1.077)**	1(1.228)**	——	24(0.934)**	11(0.744)***
Clinic	3(1.074)***	——	——	5(0.909)**	25(0.854)***	11(0.665)***
Community Hospital	4(1.186)***	10(1.077)*	——	——	1(2.279)***	5(0.093)***
Special Hospital	20(0.97)*	2(0.835)***	7(0.869)***	——	1(1.525)***	2(2.197)***
General Hospital	8(0.902)***	25(0.844)***	1(2.173)***	1(1.646)***	——	1(4.874)***
3A Hospital	25(0.842)***	24(0.711)***	25(0.591)***	4(2.001)***	5(3.757)***	——

*Note*: Taking “2(1.077)***” as an example, “2” is the bandwidth size, “1.077” is the GCLQ value, “***” indicates significance at the 1% level, and “**” indicates significance at the 5% level. Highlighted areas indicate GCLQ values greater than 1

Only one two-way colocation is detected between a pair of resources from the basic and professional categories. Community hospitals and general hospitals are spatially dependent, as indicated by the community → general GCLQ value of 2.279 and a general → community GLCQ value of 2.173. These relatively high values indicate the proximal interaction between community-level and city-level (general) hospitals. Both community hospitals and general hospitals are public institutions administered by local governments, and their spatial associations are taken into consideration by planners. In addition, China’s current resident medical insurance system (including the basic medical insurance system for urban workers, the medical insurance system for urban residents, and the new rural cooperative medical care system) requires residents to seek medical treatment in designated hospitals. Both community and general hospitals dominate the designated hospital list for most residents [63, 68]. This will affect the behaviour of residents and the location strategy for medical resources at this level.

Finally, there is a weak spatial association between all pairs of resources across the two categories (except community and general hospitals), as shown by GCLQ values of less than 1. This might reflect the disparate locations of city- and community-level services.

#### Local colocation quotient

The global colocation quotient (GCLQ) measures the direction and intensity of the global spatial associations between medical resources within and between categories across the study area but does not consider spatial heterogeneity (nonstationary colocation). The power of the LCLQ is its ability to map the direction and intensity of local spatial associations, i.e., colocation within a local neighbourhood. Interested readers can download and use this program, which was provided by Wang [31, 36]. He developed a program coded in C# to implement calibration of the aforementioned LCLQ and corresponding statistical test.

**Fig. 6:**
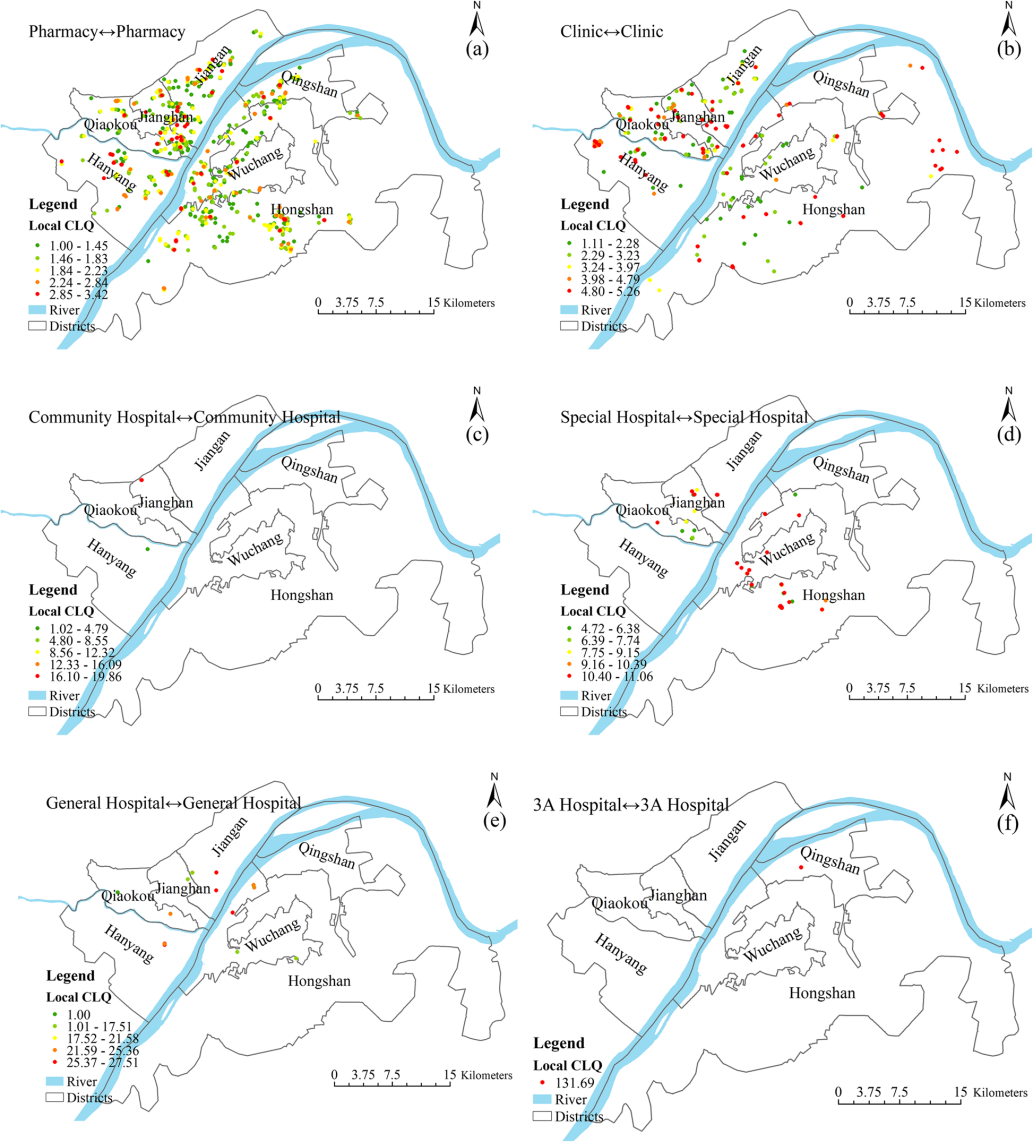
Local allocation quotient values between the six medical resources

The geographically weighted (local) colocation quotient results for all seven types of medical resources are shown in Fig. 6. Only points with a *P* value of less than 0.05 and an LCLQ value greater than 1 are mapped (Fig. 6). There is a very strong spatial association between pharmacies, as indicated by the prevalence of red and orange points (Fig. 6a). Although there is a certain degree of competition between pharmacies in terms of service objectives, the pattern is dominated by a spatial agglomeration effect. This finding provides important empirical evidence of the spatial layout of pharmacies. Figure 6b shows that although there is also a significant spatial association between clinics, the spatial agglomeration is not significant. There are 392 community hospitals in the central urban area of Wuhan, only two of which show a local spatial association; this indicates a weak spatial association between community hospitals. The main reason is that community hospitals are public medical resources, and their spatial distribution is strictly controlled by the government (Fig. 6c). The spatial association among special hospitals is strong, with LCLQ values greater than 4.5 for most of them (Fig. 6d). The intensity of spatial associations between general hospitals is lower than that of special hospitals, and the areas of spatial clustering are mainly distributed in the old town areas of Hankou and Hanyang (Fig. 6e). On the whole, the spatial association degree of the 3A hospitals is weak (Fig. 6f). The main reason is that, on the one hand, 3A hospitals have been strictly reviewed by the National Health Commission. Furthermore, 3A hospitals have absolute advantages over specialized hospitals in terms of medical equipment and doctors and have formed unique advantages in their respective medical fields, which makes the local space dependence between 3A hospitals weak. On the other hand, most of the third-class hospitals belong to the state. When choosing the sites of third-class hospitals, the government will set up third-class hospitals relatively evenly to ensure that residents can enjoy fair medical resources, which also makes the spatial dependence of third-class hospitals weak.

**Fig. 7:**
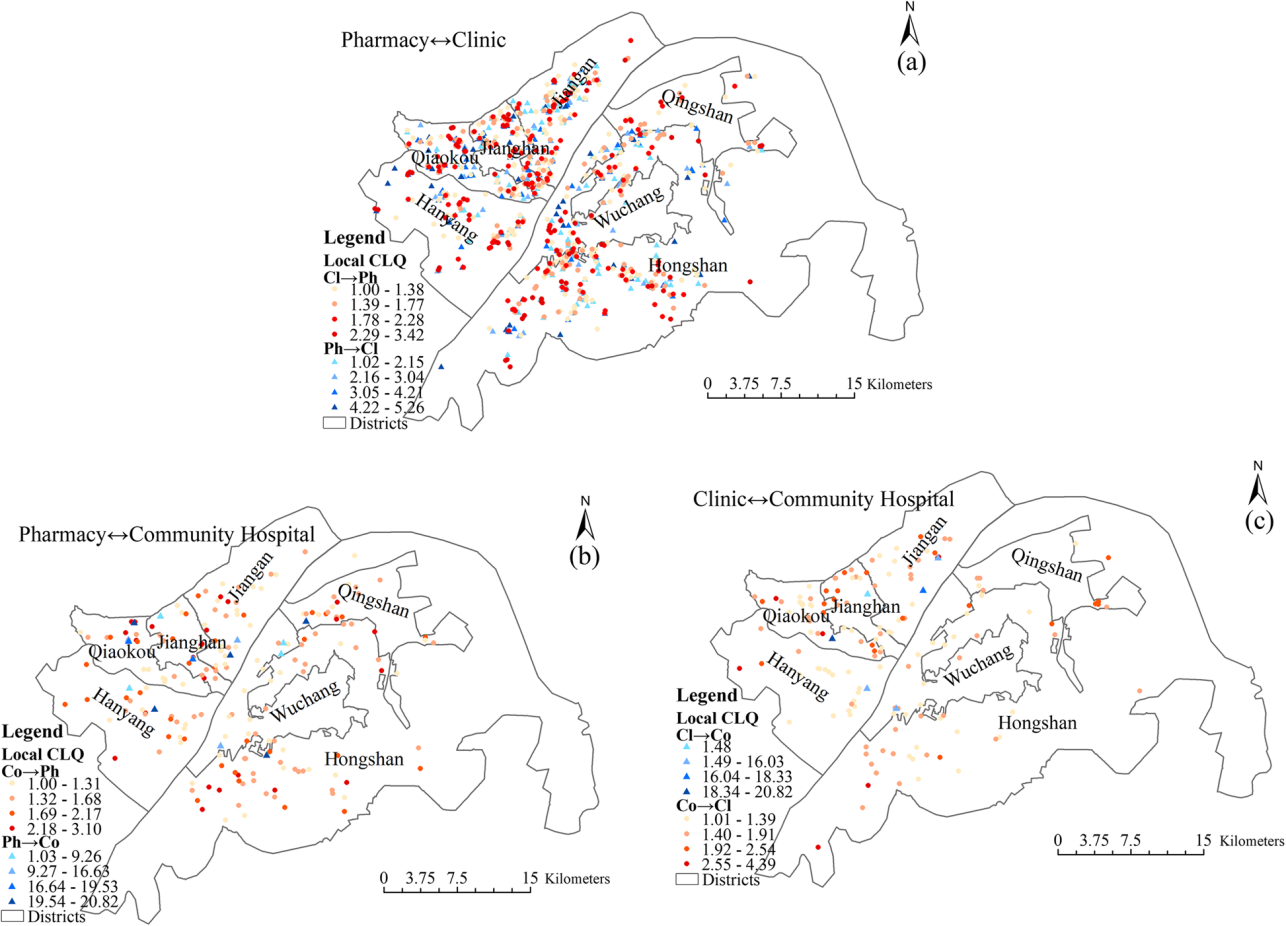
Spatial distribution of local colocation quotient values between the three types of basic medical resources (clinics, pharmacies and community hospitals)

The local colocation quotient results for basic medical resources (pharmacies, clinics and community hospitals) are shown in Fig. 7. Only points with an LCLQ value greater than 1 and a *P* value less than 0.05 are included.

The results show that pharmacies and clinics are significantly more interdependent than other medical resources (Fig. 7a). Community hospitals depend on pharmacies, but only to a small degree, as indicated by the LCLQ values of between 1 and 3.42 (Fig. 7b). Pharmacies are well distributed around the community to sell over-the-counter drugs, whereas community hospitals mainly provide medical services for designated communities. The construction and location of community hospitals are decided by government planners based on the accessibility and equity of residents’ medical treatment. Therefore, the spatial association of community hospitals on pharmacies is small. In addition, the number of pharmacies that have a spatial association with community hospitals is small, but the degree of association is large, with LCLQ values between 1 and 20.82. This is because the majority of pharmacies serving community residents are widely distributed around the community. A small number of pharmacies are dependent on community hospitals, as they provide professional auxiliary medical services for community hospitals or are recommended by community hospitals. Therefore, these pharmacies have a high degree of association with community hospitals. Third, community hospitals generally rely on clinics (Fig. 7c). However, due to the strong competitive relationship between clinics and community hospitals and the fact that clinics are smaller than community hospitals in terms of facilities and scale, most clinics do not rely on community hospitals.

**Fig. 8:**
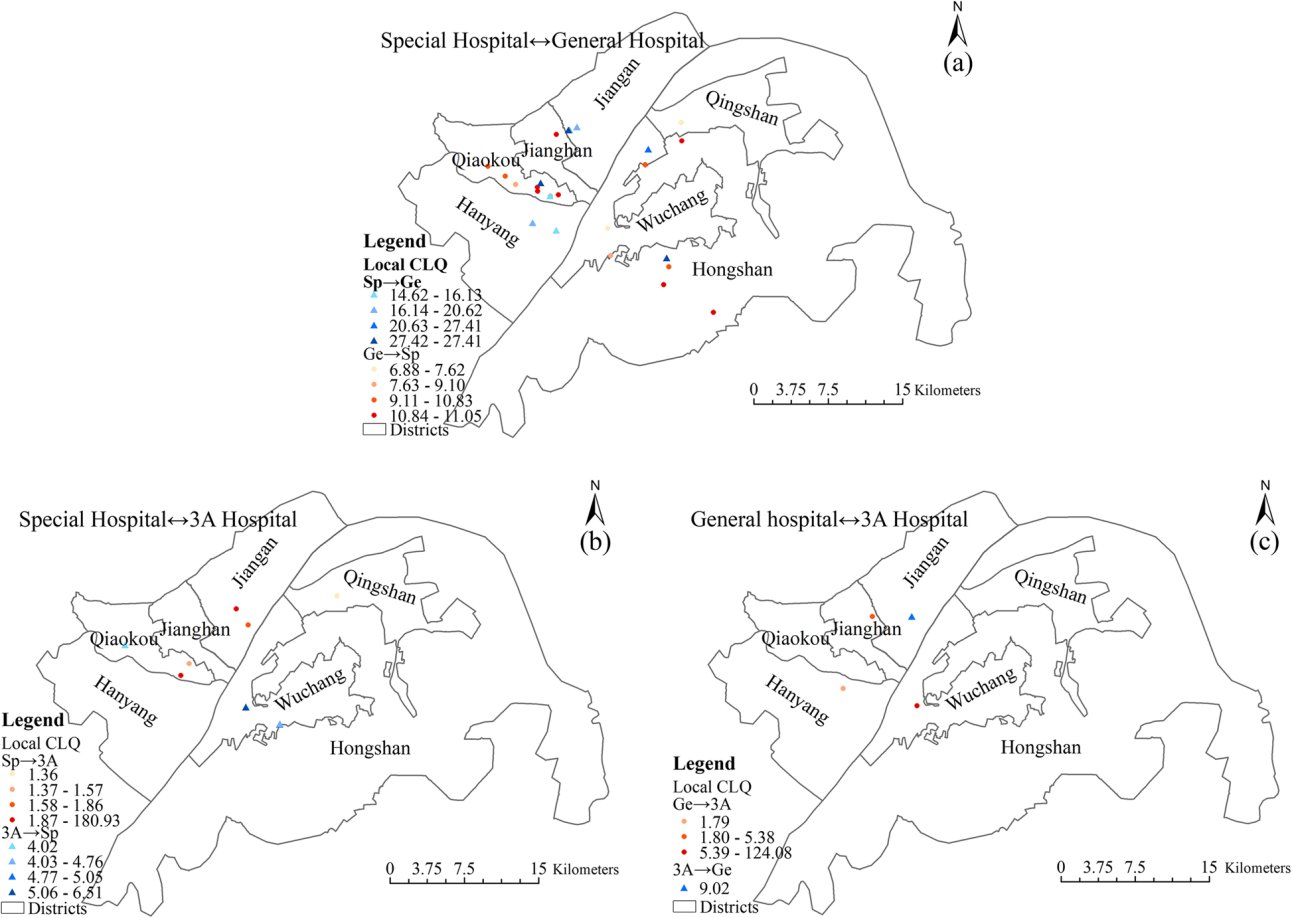
Spatial distribution of local colocation quotient values between general hospitals, special hospitals and 3A hospitals (specialist hospital, general hospital, 3A hospital)

The LCLQ values for professional medical resources (special hospital, general hospital, 3A hospital) are shown in Fig. 8, which includes only points with a *P* value of less than 0.05.

Special hospitals have a strong spatial association with general hospitals. The LCLQ values range from 14.62 to 27.42 (Fig. 8a). The spatial association of special hospitals with 3A hospitals was mainly concentrated in the vicinity of Tongji and Xiehe Hospitals. Tongji Hospital and Union Hospital have a good reputation, and patients from all over the country seek medical treatment in these locations (Fig. 8b). For this reason, many special hospitals are attached to Tongji Hospital and Union Hospital, combining their specialties to offer targeted medical services.

General hospitals have a strong spatial association with 3A hospitals (Fig. 8c). One reason is that China’s medical insurance system requires patients who need hospitalization to go to designated hospitals for treatment. Patients who cannot be treated in a general hospital are sent to a designated 3A hospital. Additionally, the Wuhan Municipal Government has issued regulations that all 3A hospitals in the city provide assistance to general hospitals, and some general hospitals are affiliated with 3A hospitals [69]. Therefore, general hospitals have a strong spatial association with 3A hospitals.

3A hospitals are less dependent on the location of special hospitals and general hospitals, although this finding is based on a relatively small sample of 45 hospitals in the central urban area of Wuhan. In addition, 3A hospitals have more specialized equipment and doctors than special hospitals or general hospitals, and they offer different services, which forms a distinct agglomeration and competition effect. Therefore, it is difficult for special hospitals and general hospitals to compete with them. Accordingly, 3A hospitals have a weak spatial association with special hospitals and general hospitals (Fig. 8).

The abovementioned results highlight the stronger local spatial association between basic medical resources and professional medical resources.

## Conclusions

This paper has analysed the directional spatial associations between six medical resources across Wuhan city using a geographically weighted colocation quotient method. This approach is particularly useful for point categorical data. Using POI data from 2019, this study has generated the following empirical evidence:

1. From the perspective of spatial distribution, pharmacies present “multicentre + multicircle” morphological distribution characteristics, clinics present a spatial pattern of “centre + axis + point”, and community hospitals present a banded distribution. Compared with other medical resources, the spatial distribution of community hospitals is more balanced [51]. Special hospitals show only one high-density hubei in Hongshan district. General hospitals are concentrated in high-density areas in Wuchang and Hankou, presenting a “dual-core” model. The 3A hospitals are concentrated in the three old urban areas of Jiang’an district, Jianghan district and Qiaokou district, while other new urban districts see less distribution [70]. In addition, in terms of spatial distribution equilibrium, pharmacies are concentrated in Jianghan district, specialized hospitals are mainly distributed in Qingshan district, and clinics are gathered in Qiaokou district. Community hospitals, general hospitals and 3A hospitals belong to public medical resources, and their spatial distribution is relatively balanced. By contrast, pharmacy, clinic, community hospital, special hospital, general hospital and 3A hospital demonstrate a certain degree of clustering or agglomeration.
2. From the perspective of spatial associations, pharmacies and clinics are significantly more interdependent than other medical resources. Community hospitals depend on pharmacies, but only to a small degree. Special hospitals have a strong spatial association with general hospitals. General hospitals have a strong spatial association with 3A hospitals. 3A hospitals are less dependent on the location of special hospitals and general hospitals. There are two networked communities of medical resources characterized by strong interactive colocations within each system: first, pharmacies, clinics, and community hospitals (local services); and second, general, specialist and 3A hospitals (city-level services). The spatial pattern of the two systems (basic and professional) reflects the history, investment and management of medical resources, as well as technical skills, medical equipment and human resource specialism, in Chinese cities [71, 72]. One strong symmetrical association between the two systems (community and general hospitals) can be detected. This indicates the linkage between medical resources at the local and city levels. The colocation association reflects the transition from local to city-level hospitals in some cases [73–75]. Although the medical resources within each category (basic and professional) demonstrate a certain degree of spatial association, the two systems of medical resources are spatially mutually exclusive.

This paper had some limitations. The POI datasets did not include all health care medical resources, such as moxibustion, foot therapy, massage, spas or sweat steaming, which might form a third system of medical resources at the neighbourhood level. In this study, the distance between points was measured using a direct-line Euclidean distance; however, in reality, people’s behaviour is more accurately predicted by transport distance, time and cost. Consequently, this approach is recommended as the measure of distance in future studies. Furthermore, the impacts of urban morphology on public service seeking behaviour should be evaluated in more detail.

## Data Availability

The datasets generated and analysed during the current study are not publicly available due to participant anonymity issues. The dataset can be made available from the corresponding author on reasonable request. A toolkit for measuring global colocation quotient (GCLQ) is downloadable at the webpage: https://ux1.eiu.edu/~bjkronenfeld/projects/. The localized colocation quotient (LCLQ) is available from the Colocation Analysis tool under the toolbox of Spatial Statistics in ArcGIS Pro 3.0. The technical process of LCLQ is explained on the following website: https://pro.arcgis.com/en/pro-app/latest/tool-reference/spatial-statistics/colocationanalysis.htm.

## Abbreviations

LCLQ: Local indicator of colocation quotient
GCLQ: Global indicator of colocation quotient
GIS: Geographic Information System
POI: Point of Interest

## Footnotes

1 1 3A hospitals are the highest level in China's classification of medical resources.
